# A Descriptive Analysis of Hybrid Cannulated Extracorporeal Life Support

**DOI:** 10.3390/jpm14020179

**Published:** 2024-02-05

**Authors:** Sebastian D. Sahli, Alexander Kaserer, Julia Braun, Raed Aser, Donat R. Spahn, Markus J. Wilhelm

**Affiliations:** 1 Institute of Anesthesiology, University and University Hospital Zurich, 8091 Zurich, Switzerland; sebastian.sahli@usz.ch; 2 Epidemiology, Biostatistics and Prevention Institute, Departments of Epidemiology and Biostatistics, University of Zurich, 8057 Zurich, Switzerland; julia.braun@uzh.ch; 3 Clinic for Cardiac Surgery, University Heart Center, University and University Hospital Zurich, 8091 Zurich, Switzerland; raed.aser@usz.ch (R.A.); markus.wilhelm@usz.ch (M.J.W.); 4 Formerly, Institute of Anesthesiology, University and University Hospital Zurich, 8091 Zurich, Switzerland; donat.spahn@swisspbm.ch

**Keywords:** extracorporeal circulation, ECMO, ECLS, mortality, outcome

## Abstract

Background: Extracorporeal life support (ECLS) is pivotal for sustaining the function of failing hearts and lungs, and its utilization has risen. In cases where conventional cannulation strategies prove ineffective for providing adequate ECLS support, the implementation of an enhanced system with a third cannula may become necessary. Hybrid ECLS may be warranted in situations characterized by severe hypoxemia of the upper extremity, left ventricular congestion, and dilatation. Additionally, it may also be considered for patients requiring respiratory support or experiencing hemodynamic instability. Method: All hybrid ECLS cases of adults at the University Hospital Zurich, Switzerland, between January 2007 and December 2019 with initial triple cannulation were included. Data were collected via a retrospective review of patient records and direct export of the clinical information system. Results: 28 out of 903 ECLS cases were initially hybrid cannulated (3.1%). The median age was 57 (48.2 to 60.8) years, and the sex was equally distributed. The in-hospital mortality of hybrid ECLS was high (67.9%). In-hospital mortality rates differ depending on the indication (ARDS: 36.4%, refractory cardiogenic shock: 88.9%, cardiopulmonary resuscitation: 100%, post-cardiotomy: 100%, others: 75%). Survivors exhibited a lower SAPS II level compared with non-survivors (20.0 (12.0 to 65.0) vs. 55.0 (45.0 to 73.0)), and the allogenic transfusion of platelet concentrate was observed to be less frequent for survivors (0.0 (0.0) vs. 1.8 (2.5) units). Conclusion: The in-hospital mortality rate for hybrid ECLS was high. Different indications showed varying mortality rates, with survivors having lower SAPS II scores and requiring fewer platelet concentrate transfusions. These findings highlight the complexities of hybrid ECLS outcomes in different clinical scenarios and underline the importance of rigorous patient selection.

## 1. Introduction

Extracorporeal life support (ECLS) plays a critical role in cardiopulmonary assistance once conventional measures have been exhausted. It has become a key support instrument during organ recovery or the onset of destination therapy [1,2]. Veno-venous (V-V) and veno-arterial (VA) ECLS modifications are standard. If oxygenation or ventilation is predominantly impaired, support is provided by passing venous blood through an artificial lung membrane. The afferent limb returns the decarboxylated and oxygenated blood back to the venous system. If a patient has cardiovascular disease and consequent hemodynamic instability, the blood from the membrane lung can be returned to the arterial system. In addition to oxygenation, it provides a pumping function to maintain perfusion pressure. Specific limitations such as age, malignancy, or patient preference, as well as structural and staffing requirements, must be considered [3].

During ECLS therapy, the supply of oxygenated blood to the coronary arteries and brain is dependent on pulmonary function (V-V) or retrograde aortal flow (VA) [4]. However, a modified system with an additional third (sometimes even fourth) cannula is required in some cases in which ineffective ECLS support presents with a mixed picture of hypoperfusion and hypoxemia due to competing or insufficient circuits [5]. Unfortunately, only few data are available on the adaption of the ECLS toward a hybrid cannulation [6,7,8,9,10,11,12]. According to the Extracorporeal Life Support Organization (ELSO) report 2017, 2% of adults supported with ECLS for cardiac failure and less than 1% on ECMO for respiratory failure are managed with a hybrid cannulation [13]. The analysis of the Chinese Extracorporeal Life Support Registry (CSECLS) showed 19 (0.6%) initial hybrid configurations out of 3102 adult ECLS cases between 2017 and 2019 [6].

In recent years, there has been increasing awareness of the need for a standardized nomenclature for ECLS therapy. The use of standardized descriptions of the ECLS modification allows for comparison and analysis (also from center to center). For this purpose, the ELSO position papers provide concrete rules for the ECLS nomenclature [14,15]. The use of a hyphen to differentiate drainage cannulas to the left of the hyphen from return cannulas to the right of the hyphen is the core of any cannulation abbreviation. The hyphen itself symbolizes the membrane lung (ML). According to the Maastricht Treaty for ECLS nomenclature [14], the third added cannula is defined by an additional capital letter placed outside the existing cannulas. For example, if a second venous drain cannula is added, the letter “V” is placed to the left of the hyphen and outside the first drain cannula, resulting in “VV-A”. In a V-A setup, if an additional re-entry cannula is inserted alongside the initial arterial cannula to improve systemic oxygenation, the letter “V” is added to the outer right to indicate post-ML flow (return): “V-AV”. The insertion of an arterial cannula into a “V-V” configuration is indicated as “V-VA”. Consequently, the order of configurations during ECLS therapy reflects the patient’s temporal support needs and conveys a chronological sequence. But the nomenclature lacks differentiation between V-V and V-A proportional support. And from a physiological standpoint, V-VA is indistinguishable from V-AV. Besides the cannula hierarchy and localization, the position of the cannula tip and its dimensions are also relevant.

The hybrid ECLS configuration supports patients with combined cardiopulmonary failure who cannot be successfully assisted with the conventional veno-venous or veno-arterial alone. Hybrid ECLS may be required in conditions of severe hypoxemia of the upper extremity, also known as Harlequin syndrome or north–south syndrome [2], but also in cases of left ventricular congestion and dilatation [5]. A supplemental inflow cannula can be placed in the internal jugular vein to deliver oxygenated blood to the pulmonary circulation. This helps correct differential hypoxemia by directing oxygenated blood back to the right ventricle then through the pulmonary circulation, left ventricle, and out to the coronary arteries and aortic arch vessels [16]. Another relevant indication includes ECMO patients receiving respiratory support who also exhibit concurrent hemodynamic instability, particularly in cases of right heart failure [5] but also in left or biventricular failure. For hemodynamic support, an arterial perfusion cannula is inserted into the circuit, usually via the femoral or subclavian artery. 

This study investigates the factors that determine the association between hybrid ECLS and patient outcomes. We analyzed all initially hybrid cannulated ECLS cases between the period from 2007 to 2019 at the University Hospital of Zurich.

## 2. Materials and Methods

### 2.1. Study Design 

All hybrid ECLS cases of adults at the University Hospital Zurich (USZ) in Switzerland between January 2007 and December 2019 with initial triple cannulation were included. We differentiated V-AV (efferent limb venous; afferent arteriovenous) and VV-A (efferent limb veno-venous; afferent arterial) configuration. Modifications at a later stage of V-A ECLS and V-V ECMO were not included. Further exclusion criteria were age below 18 years and documented refusal of general consent. We grouped indications for hybrid ECLS therapy into five common categories according to the current literature [1,2,6,8]: “acute respiratory distress syndrome”, “refractory cardiogenic shock”, “cardiopulmonary resuscitation”, “post-cardiotomy”, and “other”. 

The study was approved, and the requirement for written informed consent was waived by the Cantonal Ethics Commission of Zurich, Switzerland (BASEC-Nr. 2019-01926).

### 2.2. Assessment of Hybrid Modification

Based on the underlying disease, the indication was for either V-V or V-A ECLS therapy. In cases where the medical team, consisting of a cardiac anesthesiologist or intensivist, a cardiac surgeon, and a perfusionist, identified inadequate drainage or perfusion during initialization of the baseline configuration, a hybrid procedure was adapted. As recommended in the ELSO nomenclature, when a VVA mode configuration is implemented primarily for cardiac support, it is expressed as V-AV (initial indication V-A).

### 2.3. Study Endpoints 

We recorded the in-hospital mortality to compare survivors and non-survivors. Furthermore, 1-year mortality was assessed.

### 2.4. Data Collection and Variables 

Data were collected via a retrospective review of the medical records of all included patients with hybrid ECLS (medical history, last laboratory values, ECLS configuration and duration, complications, length of ICU stay, and outcomes) and direct export of the clinical information system via medical controlling (age, gender, number of transfused red blood cells, fresh frozen plasma, and platelet concentrate, and length of hospital stay). 

### 2.5. Statistical Analysis 

For descriptive statistics, we show the median and interquartile range (IQR 25% to 75%) for continuous variables. For categorical variables, we show counts (n) and proportions (%). Due to the small sample size, we chose not to present statistical tests, as this would quickly lead to a multiple-testing problem, which in turn would make any meaningful statistical interpretation impossible.

## 3. Results

**Figure 1:**
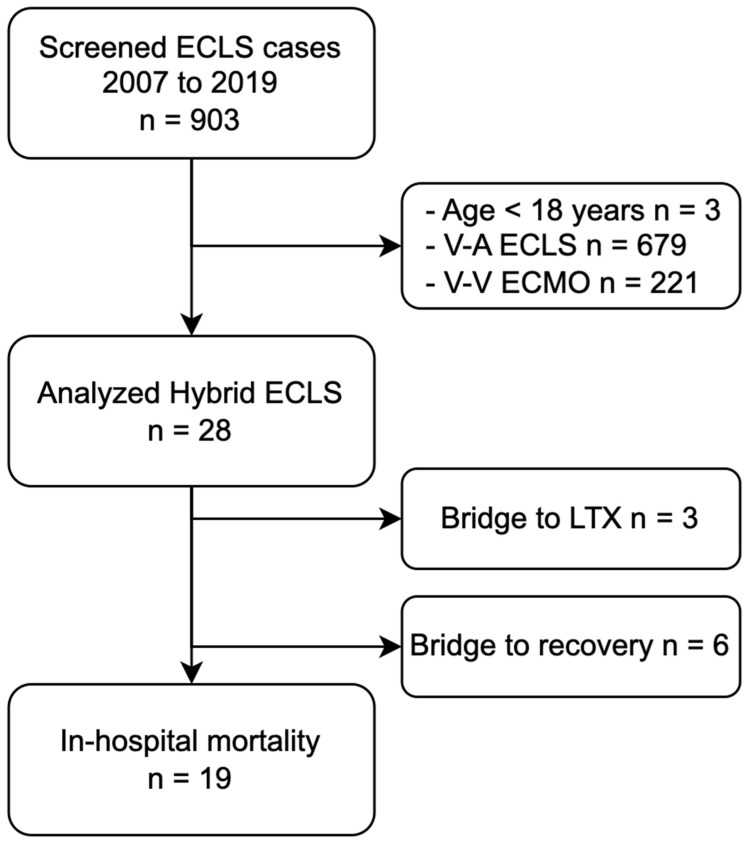
Flowchart of patient selection and hybrid ECLS outcome. Abbreviations: ECLS, extracorporeal life support; ECMO, extracorporeal membrane oxygenation; LTX, lung transplantation; V-A, veno-arterial; V-V, veno-venous.

We screened 903 ECLS cases between 2007 and 2019, 28 of whom (3.1%) were instances of initial hybrid ECLS cannulation and matched the inclusion criteria (Figure 1). 

### 3.1. Patient Characteristics and Mortality

The median age was 57 (48.2 to 60.8) years, and the sex was equally distributed. The most frequent comorbidity at the time of ECLS cannulation was coronary artery disease (17.9%). Patients had a high SAPS II within the first 24 h of ICU admission, and survivors exhibited a lower SAPS II level compared with non-survivors (20.0 (12.0 to 65.0) vs. 55.0 (45.0 to 73.0)) (Table 1). Fifteen patients died during hybrid ECLS therapy, and four patients died after weaning. Overall, the in-hospital mortality was high (19 out of 28, 67.9%) (Table 2).

### 3.2. Outcomes of Survivors

Six patients were successfully weaned, and three patients had bridged-to-lung transplantation. Except for two patients who were lost to follow-up, all patients were alive at one year (Table 3).

### 3.3. Hybrid ECLS Indication

**Figure 2:**
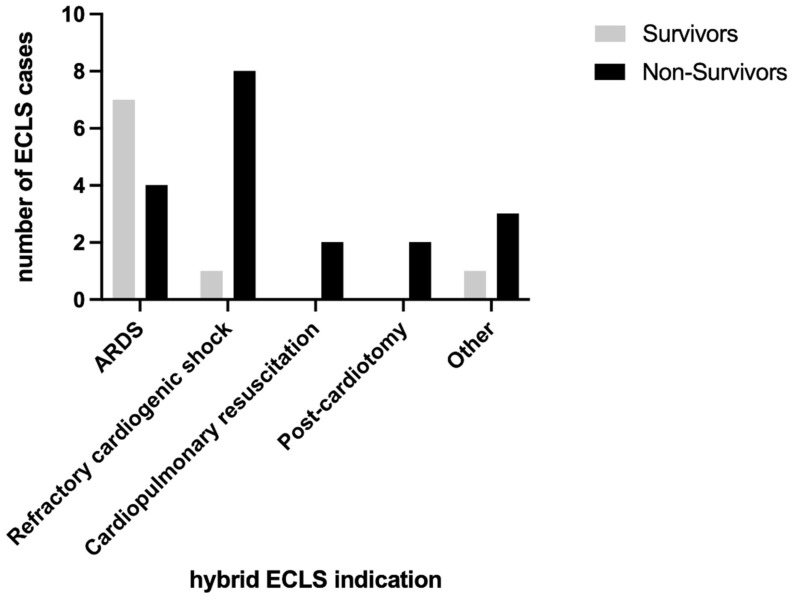
Survivors and non-survivors stratified by indication of hybrid ECLS.

The largest group of hybrid ECLS indications was represented by patients suffering from ARDS who showed a higher survival at hospital discharge (7 out of 11 cases), resulting in an in-hospital mortality of 36.4% (Figure 2). Patients with refractory cardiogenic shock showed a high in-hospital mortality of 88.9% (eight out of nine). Patients with hybrid ECLS indications for cardiopulmonary resuscitation and post-cardiotomy showed 100% in-hospital mortality (two out of two each). The indication “other” consisted of two patients suffering from acute respiratory insufficiency (not defined as ARDS), one patient with pulmonary scleroderma, and one patient diagnosed with obstructive shock due to an atrial tumor. Only one of the four patients in the “other” group survived, resulting in an in-hospital mortality of 75%.

### 3.4. Hybrid ECLS Cannulation Details

**Figure 3:**
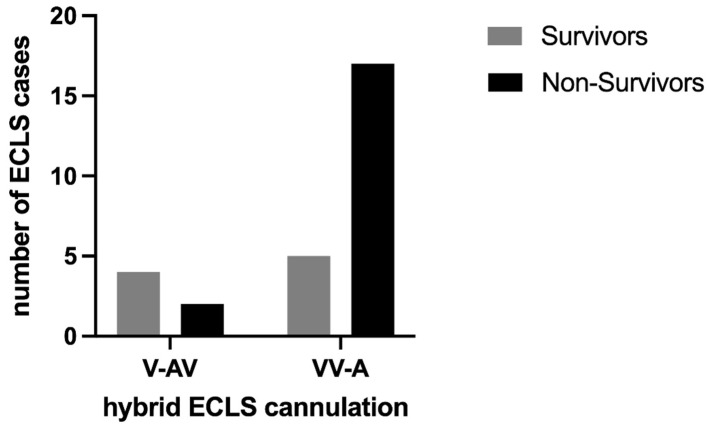
Survivors and non-survivors stratified by cannulation of hybrid ECLS.

**Figure 4:**
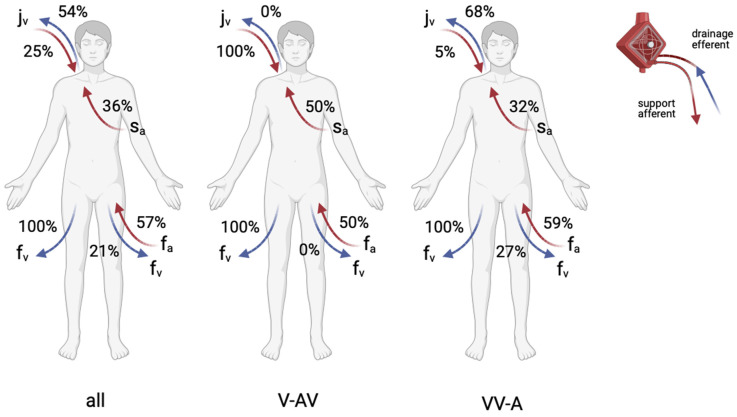
Cannulation sites for hybrid configuration according to frequency. (All) All hybrid ECLS cannulation percentages together, (V-AV) venous-arteriovenous hybrid modification (6 cases), and (VV-A) venovenous-arterial hybrid modification (22 cases). The cannulation site of one case (efferent right atrium and afferent pulmonary artery, 3.6% each) is not shown due to visibility. The right or left site is arbitrary. Note that the sum of percentages is equal to 300% due to three cannulation sites each. Abbreviations: j_v_ internal jugular vein; s_a_ subclavian artery; f_v_ femoral vein; f_a_ femoral artery. Created with BioRender.com, accessed on 9 December 2023, with confirmation of publication and licensing rights.

Most cannulations were performed peripherally using the Seldinger technique (71.4%). Six ECLS cases were performed as V-AV configurations and the others as VV-A (Figure 3). Regarding the efferent ECLS limb, a venous femoral drain was installed in each case. The further combinations of V-AV and VV-A modifications are shown in Figure 4. The in-hospital mortality was higher in the VV-A group (17 of 22, 77.3%) compared to the V-AV group (2 of 6, 33.3%). 

### 3.5. Hybrid ECLS Complications

The allogenic transfusion of platelet concentrate was higher for non-survivors compared to survivors (1.8 vs. 0 units). Besides renal replacement therapy, major bleeding events were the most frequent complications and were also observed more frequently in the cohort of non-survivors (Table 2).

## 4. Discussion

This retrospective single-center study reports 28 initially hybrid-cannulated ECLS cases at the University Hospital of Zurich in the period from 2007 to 2019. We observed a high in-hospital mortality of 67.9% (n = 19); 4 of the 19 non-survivors died after ECLS weaning. The most frequent diagnosis at the time of hybrid ECLS installation was ARDS (11 patients), with more survivors than non-survivors in this group. Overall, non-survivors had a higher SAPS II and more frequently received transfusion of allogenic platelet concentrates.

Although only a few studies are available for hybrid ECLS with a low number of included patients (3 to 26 patients) [6,7,8,9,10,11,12,17], the in-hospital mortality observed at our institution is in line with that in recently published studies. Biscotti et al. [9] and Ius et al. [11] reported lower mortality rates of 57.1% and 50.0%, but they also included patients who were initially conventionally cannulated and only later modified to a hybrid strategy. ECLS indications in those studies were predominantly pulmonary diseases. Looking separately at the indication ARDS of our study, the in-hospital mortality is as low as 36.4%. This is in accordance with a study published in 2010 with a small number of cases [12]. A recently published retrospective study of patients with predominant ARDS on V-V ECMO support requiring a change to V-VA support showed an in-hospital mortality rate of 37% [18]. 

We recently published our outcome analysis of 679 V-A [19] and 221 V-V [20] ECLS cases. The frequency of hybrid ECLS in our institution (3%) is in line with published data of ELSO [13] (2% of adults supported for cardiac failure and less than 1% for respiratory failure). The Chinese Extracorporeal Life Support Registry (CSECLS) states that 0.6% of initial hybrid configurations between 2017 and 2019 [6]. 

The institutional comparison shows a higher in-hospital mortality rate between conventionally V-A-assisted and hybrid-assisted ECLS cases (V-A vs. hybrid: indication post-cardiotomy 70.7% vs. 100%, cardiopulmonary resuscitation 67.9% vs. 100%, refractory cardiogenic shock 47.0% vs. 88.9%). Mortality is nearly identical between V-V and hybrid-assisted patients (V-V vs. hybrid: indication ARDS 36.2% vs. 36.4%). It is likely that the sicker patients needed more support initially. In terms of selection bias, the initial hybrid cannulated patients also have a higher mortality rate. This finding should certainly be taken into account when the treatment team is considering hybrid cannulation. As compared to V-A and hybrid cannulation, V-V cannulated cases have fewer major bleeding complications (16.7%, 25.2%, and 28.6%) or leg ischemias (6.3%, 13.4%, and 10.7%) at our institution. Complication rates are similar between V-A and hybrid. But the small number of hybrid cannulated cases must be considered.

In our study, we describe three patients who were treated with a hybrid ECLS bridging procedure and who were finally treated with a lung transplantation. Bridging to heart transplantation was not observed. It is crucial to note that ECLS therapy is not a definitive treatment but rather a bridge to recovery in the context of significant lung or heart failure, as well as a bridge to decision-making. ECLS often necessitates high doses of anticoagulation, posing a risk of bleeding, particularly intracranial, and requiring massive transfusion [21]. This is associated with adverse effects on patient morbidity and mortality [22], not to mention the additional immunologic effects and increased risk of a positive cross-match in planned transplants. On the positive side, advancements in treatment protocols and expertise in specialized centers over the past decade have made bridging to lung transplantation feasible for patients with end-stage lung disease, yielding reasonable outcomes [23]. Apart from optimized gas exchange, these patients experience reduced sedation requirements and less confinement to the hospital bed, allowing for better-organized physiotherapy and nutrition. While pharmacological treatments for end-stage heart failure have improved, the majority of patients succumb to the disease, with heart transplantation being the sole option [24]. However, the scarcity of donor organs has led to the establishment of ECLS as the primary alternative, serving as a bridge to recovery or as a bridge-to-bridge, allowing for the subsequent implantation of a long-term device, or as a bridge-to-transplant [25]. It is important to recognize ECLS as a high-risk procedure with significant morbidity and mortality. Additionally, the limited availability of healthcare resources and ethical considerations must be taken into account.

The cannulation strategy depends on the underlying pathology and is implemented in various ways. Nevertheless, principles are now emerging to improve systemic oxygenation or cardiac unloading [26,27]. A V-VA configuration has been proposed in patients with differential hypoxia (Harlequin syndrome) or secondary heart failure (e. g., right ventricular impairment) after V-V ECMO initiation [5]. We describe six cases with an in-hospital mortality of 33.3%. The other cannulation mode, VV-A, combines the need for higher venous drainage with the possibility of providing both circulatory and respiratory support and is more complex [5]. Interestingly, this cannulation strategy is predominant in our study. Their markedly higher in-hospital mortality of 77.3% reflects this statement.

Besides renal replacement therapy, major bleeding events were the most prevalent complication during hybrid ECLS therapy. With a frequency of 28.6%, major bleeding occurred less frequently than described in the literature, with almost every second patient suffering from major bleeding [8]. In contrast to our expectation, complications like leg ischemia and intracranial bleeding were rare in our cohort. Werner et al. [7] and Mihu et al. [8] reported higher incidences of these complications, despite a conservative anticoagulation regimen. As a possible reason, they mention that more than half of their patients were externally cannulated and that there was no radiological imaging of the brain prior to initiation of ECLS. A patient-oriented balance needs to be struck between the postulated benefits of hybrid cannulation and the potential harm it may cause. There is an increased risk of bleeding, infection, and thrombosis. Additional technical problems arise, such as the difficulty of flow measurement with parallel systems, as well as their flow regulation.

Retrograde aortic flow may lead to impaired myocardial function and pulmonary congestion as an inherent disadvantage of VA treatment. In addition to hybrid cannulation, another strategy to improve hemodynamics in cardiogenic shock is venting [28]. The first systematic review of left ventricular unloading with Impella in addition to VA-ECMO (“ECMELLA”) described an improved survival and neurological outcome despite higher complication rates compared to VA-ECMO alone [29].

## 5. Conclusions

The in-hospital mortality rate for hybrid ECLS was high. Different indications showed varying mortality rates, with survivors having lower SAPS II scores and requiring fewer platelet concentrate transfusions. These findings highlight the complexities of hybrid ECLS outcomes in different clinical scenarios and underline the need for rigorous patient selection.

## 6. Limitations

The small sample size of the study presents challenges in generalizing the results. Results from a single high-volume ECMO center may have limited applicability to broader populations. In addition, the inclusion of patients over an extended period of time includes the potential for changes in both the patient population and standards of care that may have evolved over time. Finally, the analysis focuses on a heterogeneous cohort, reflecting the inherent complexity of this specialized therapy. Despite these limitations, retrospective studies provide valuable insights into associations between variables and serve as a basis for generating hypotheses that merit further investigation.

## Figures and Tables

**Table 1:** Characteristics of hybrid ECLS patients stratified for survivors and non-survivors.

	All	Survivors	Non-Survivors
	[n = 28]	[n = 9]	[n = 19]
**Patient characteristics**			
Age (years)	57.0 (48.2 to 60.8)	55.0 (32.0 to 57.5)	57.0 (49.0 to 64.0)
BMI (kg/m^2^)	25.5 (22.7 to 33.0)	30.1 (22.1 to 36.2)	24.7 (22.5 to 31.2)
Sex (female)	14 (50.0)	4 (44.4)	10 (52.6)
SAPS II (points)	53.5 (29.3 to 68.0)	20.0 (12.0 to 65.0)	55.0 (45.0 to 73.0)
Charlson comorbidity index	2.5 (1.0 to 3.8)	2.0 (1.0 to 3.5)	3.0 (1.0 to 4.0)
**Comorbidities**			
Coronary artery disease	5 (17.9)	1 (11.1)	4 (21.1)
Congestive heart failure	3 (10.7)	0 (0.0)	3 (15.8)
Peripheral vascular disease	2 (7.1)	0 (0.0)	2 (10.5)
Obstructive pulmonary disease	3 (10.7)	0 (0.0)	3 (15.8)
Diabetes mellitus	3 (10.7)	0 (0.0)	3 (15.8)
Chronic kidney disease	0 (0.0)	0 (0.0)	0 (0.0)
**Baseline laboratory parameters**			
pH	7.273 (7.140 to 7.455) (7)	7.273 (7.121 to 7.392)	7.282 (7.132 to 7.458)
pO_2_ (kPa)	7.9 (7.1 to 12.1) (7)	8.1 (7.1 to 22.7)	7.9 (6.9 to 12.1)
pCO_2_ (kPa)	5.8 (4.6 to 8.6) (7)	7.7 (5.7 to 12.9)	5.6 (4.4 to 8.4)
Lactate (mmol/L)	2.5 (1.5 to 6.9) (7)	1.6 (1.2 to 4.4)	2.9 (1.5 to 10.1)
Hemoglobin (g/L)	89.5 (79.3 to 102.5) (0)	86.0 (77.0 to 102.0)	92.0 (80.0 to 112.0)
Myoglobin (µg/L)	164.0 (58.0 to 536.0) (5)	175.0 (101.3 to 334.3)	164.0 (48.0 to 859.0)
Creatinine (µmol/L)	115.0 (78.0 to 171.0) (1)	109.0 (73.5 to 142.0)	127.0 (78.3 to 196.5)

Data presented as median and interquartile range (IQR). Categorical variables as number and percentage (%). If necessary, missing data are indicated in parentheses [n]. Abbreviations: BMI, body mass index; ECLS, extracorporeal life support; SAPS II, simplified acute physiology score II.

**Table 2:** Description of hybrid ECLS cases stratified for survivors and non-survivors.

	All	Survivors	Non-Survivors
	[n = 28]	[n = 9]	[n = 19]
**ECLS**			
Indication			
ARDS	11 (39.3)	7 (77.8)	4 (21.1)
Refractory cardiogenic shock	9 (32.1)	1 (11.1)	8 (42.1)
Cardiopulmonary resuscitation	2 (7.1)	0 (0.0)	2 (10.5)
Post-cardiotomy	2 (7.1)	0 (0.0)	2 (10.5)
Other	4 (14.3)	1 (11.1)	3 (15.8)
ECLS outcome			
Successful weaning	9 (32.1)	6 (66.7)	3 (15.8)
Bridge to assist device	1 (3.6)	0 (0.0)	1 (5.3)
Bridge to lung transplantation	3 (10.7)	3 (33.3)	0 (0.0)
**Complications During ECLS Therapy**			
Transfusions			
Red blood cells (units)	4.0 (2.0 to 7.8)	4.0 (2.5 to 6.5)	4.0 (1.0 to 8.0)
	6.8 (9.0)	8.3 (13.1)	6.0 (6.5)
Fresh frozen plasma (units)	0.0 (0.0 to 1.0)	0.0 (0.0 to 1.0)	0.0 (0.0 to 1.0)
	0.6 (1.0)	0.3 (0.5)	0.7 (1.2)
Platelet concentrate (units)	0.0 (0.0 to 2.0)	0.0 (0.0 to 0.0)	1.0 (0.0 to 3.0)
	1.3 (2.2)	0.0 (0.0)	1.8 (2.5)
Major bleeding	8 (28.6)	2 (22.2)	6 (31.6)
Intracranial bleeding	1 (3.6)	0 (0.0)	1 (5.3)
Stroke	0 (0.0)	0 (0.0)	0 (0.0)
Liver failure	1 (3.6)	1 (11.1)	0 (0.0)
Renal replacement therapy	12 (42.9)	4 (44.4)	8 (42.1)
Ischemia extremities	3 (10.7)	1 (11.1)	2 (10.5)
Open chest therapy	4 (14.3)	0 (0.0)	4 (21.1)
**Duration**			
Length ECLS (days)	8.5 (3.3 to 14.3)	11.0 (8.0 to 18.5)	6.0 (2.0 to 12.0)
Length ICU (days)	19.5 (8.5 to 31.3)	26.0 (18.5 to 42.0)	13.0 (6.0 to 29.0)
Length of hospital stay (days)	26.5 (8.5 to 43.0)	32.0 (19.5 to 82.0)	23.0 (6.0 to 34.0)
**Mortality**			
In-hospital mortality	19 (67.9)	0 (0.0)	19 (100)
Death during ECLS therapy	15 (53.6)	0 (0.0)	15 (78.9)
1-year survival	7 (26.9) (2)	7 (77.8)	0 (0.0)

Data presented as median and interquartile range (IQR). For transfusion counts, mean and standard deviation (SD) are listed as well. Categorical variables as number and percentage (%). If necessary, missing data are indicated in parentheses [n]. Abbreviations: ARDS, acute respiratory distress syndrome; ECLS, extracorporeal life support; ICU, intensive care unit.

**Table 3:** Outcome of survivors.

	ECLS	Outcome	Discharge	1-y Survival
**Patient**				
1	V-AV	Successful weaning	Transfer to another hospital	Loss of follow-up
2	V-AV	Successful weaning	Rehabilitation	Yes
3	VV-A	Successful weaning	Transfer to another hospital	Yes
4	VV-A	Successful weaning	At home	Yes
5	VV-A	Successful weaning	Rehabilitation	Yes
6	VV-A	Bridge to LTX	At home	Yes
7	VV-A	Bridge to LTX	Rehabilitation	Yes
8	V-AV	Successful weaning	At home	Loss of follow-up
9	V-AV	Bridge to LTX	At home	Yes

Abbreviations: 1-y Survival, 1-year survival; ECLS, extracorporeal life support; LTX, lung transplantation; VV-A, ECLS mode venovenous-arterial; V-AV, ECLS mode veno-venoarterial.

## Data Availability

The data are not publicly available due to privacy or ethical restrictions.
